# An Evaluation of the Rabies Surveillance in Southern Vietnam

**DOI:** 10.3389/fpubh.2021.610905

**Published:** 2021-04-29

**Authors:** Quang Duy Pham, Lan Trong Phan, Thuy Phuong Thi Nguyen, Quan Minh Ngoc Doan, Hai Duc Nguyen, Quang Chan Luong, Thuong Vu Nguyen

**Affiliations:** ^1^Planning Division, Pasteur Institute in Ho Chi Minh City, Ho Chi Minh City, Vietnam; ^2^Training Center, Pasteur Institute in Ho Chi Minh City, Ho Chi Minh City, Vietnam; ^3^Vietnam Field Epidemiology Training Program, General Department of Preventive Medicine, Ministry of Health, Hanoi, Vietnam; ^4^Directorial Board, Pasteur Institute in Ho Chi Minh City, Ho Chi Minh City, Vietnam; ^5^Department for Disease Control and Prevention, Pasteur Institute in Ho Chi Minh City, Ho Chi Minh City, Vietnam

**Keywords:** rabies, surveillance, evaluation, post-exposure prophylaxis, Vietnam

## Abstract

**Introduction:** Rabies is endemic in Vietnam and has been a statutory notifiable infectious disease since 1998. We, herein, assessed the performance of rabies surveillance in Southern Vietnam and identified areas for improvement.

**Materials and Methods:** We analyzed data on human rabies cases reported during 1991–2018. We adapted guidelines from the U.S. Centers for Disease Control and Prevention to evaluate attributes of surveillance. Between June and November 2018, we interviewed a total of 145 staff from hospitals, preventive medicine centers, and animal health offices at provincial and district levels in five southern provinces.

**Results:** Between 2009 and 2018, an average of nine cases of human rabies (range: 4–20 cases) was reported annually in Southern Vietnam, representing an incidence of 2.7 cases per 10 million population. The highest incidence was observed in 2018 (5.5 cases per 10 million population). Survey data suggested that only 24% (13/53) of participants agreed that the monthly report template was easy to complete and that 42% (23/55) indicated that the change from the paper-based to the electronic case notification systems was easy. Only 7% (2/29) of human rabies cases were reported timely, and 65% (13/20) successfully collected specimens. Approximately 39% (56/144) of staff were aware of turning surveillance data into prevention activities, and 21% (31/145) witnessed data used for strategic program decision making.

**Conclusions:** Although rabies surveillance was quite simple, flexible, and accepted in southern Vietnam, simplifying the report forms, training staff, and improving the timeliness of reporting and data usage are highly recommended for a better implementation of rabies surveillance.

## Introduction

Worldwide, an estimated 59,000 people die from rabies every year, with the majority (95%) being in Africa and Asia due to the shortage of post-exposure prophylaxis (PEP) services for animal-bite victims and the lack of experienced personnel and facilities for rabies surveillance (1). This deadly disease, however, is effectively prevented by human and animal vaccinations. Vaccinating domestic dogs and cats successfully prevents the spread of the rabies virus, and immediate administration of PEP, consisting of rabies vaccine and immune globulin, effectively reduces the risk of disease for most persons with probable rabies exposures (2).

In Vietnam, 4,234 cases of human rabies deaths were reported between 1991 and 2018 (3). To reduce the spread of rabies, Vietnam strengthened its rabies control and prevention system nationwide in 1996, focusing on community engagement through communication channels, vaccination of dogs, prevention of dog bites, surveillance of canine and human rabies, coordination of multiagency activities, PEP services, and vaccine availability and research. Such enhancement was largely responsible for bringing about a decline in the incidence of the disease by 95% between 1994 and 2003 (3). However, notifications for human rabies cases have remained relatively high, with an average of 90 cases recorded each year between 2004 and 2018 (3).

Like other public health surveillance, rabies surveillance provides essential data for monitoring rabies cases in both human and dog populations, which informs decision making around prevention and responses. Rabies surveillance also helps track progress toward the global goal of eliminating human deaths from dog-mediated rabies by 2030 (2). Assessing and improving surveillance performance *via* periodic evaluations are thus important, especially in countries where human rabies remains a significant public health issue (4). In 2014, Vietnam's Ministry of Health launched its national guideline for rabies surveillance, control, and prevention after decades using internally circulating materials made by four subnational public health institutes. The new guideline provides health care facilities a clear, unified methodology and structural framework for building the system's capacity, for implementing surveillance and response activities, and for reporting throughout the country. Although this guideline has been implemented nationwide for 5 years, its impact has not been formally assessed.

Our objectives were (1) to review implementation of rabies surveillance between 2009 and 2018 (2); to analyze routinely collected surveillance data from the human health sector; and (3) to identify potential areas for improvement of both human health and animal health sector surveillance and prevention in Southern Vietnam in 2018.

## Methods

### Desk Review of Implementation of Surveillance Guidelines

To review the implementation of Vietnam's surveillance guideline for rabies over time, we used a standardized questionnaire to interview experienced surveillance staff of subnational public health institutes. We collected information about the year of publication, the authority levels of decisions, and specific surveillance areas covered by multiple surveillance documents: rabid animals, outbreaks of canine rabies, dog-bite victims, and human rabies cases.

### Review of Routinely Collected Surveillance Data

We collated and reviewed all reports of cases of human rabies from 20 provinces in the Southern Vietnam submitted to the Pasteur Institute in Ho Chi Minh City (PIHCMC) between 2009 and 2018. Southern Vietnam covers an area of 74,152 km^2^, with a population of more than 36 million people in 2018. We assessed the completeness of reporting for the 5 years from 2013 through 2017 by comparing the number of case notifications with the number of patients assigned ICD10 code A82.9 issued on September 24, 2015, at hospitals during site visits to hospitals, preventive medicine centers, and animal health offices. Site visits also included collection of data on the estimated size of dog and cat populations, canine vaccine coverage, and number of people receiving PEP for the period 2013–2017 in the assessment areas.

### Survey of Human Health and Animal Health Workers

We conducted a surveillance evaluation using standard guidelines (5). To capture variations in geography, social status, economic development, and rabies detection and response, the evaluation covered 10 districts in five provinces, which reported at least one case of human rabies during the 5 years from 2013 through 2017: Ho Chi Minh City (HCMC, the largest city in Vietnam), two provinces from the southeast region (Tay Ninh and Dong Nai), and two provinces from the southwest region (Long An and Ca Mau).

At the time of our evaluation, each province had a Provincial Center for Disease Control (PCDC), which is responsible for the implementation, management, and coordination of public health activities and infectious disease surveillance and response in the province. In the selected provinces, potential participants, who were clinicians, nurses, veterinarians, and program staff from hospitals, PCDCs, and subanimal health offices at provincial and district levels, were identified in consultation with the associated PCDCs between June and November 2018.

After being briefed about the purposes and procedures of this evaluation and receiving answers for questions from PIHCMC staff, participants gave oral consent and received 100,000 Vietnam dong (~4.5 US dollars) for their participation. To ensure confidentiality for the participants, we did not request any identifying information.

We conducted semi-structured interviews in a private room at facilities where the participants worked. The content, logic, and language of the questionnaire were reviewed before being used for interviews. The questionnaire contained 45 close-ended and open-ended questions and divided into four sections: (i) general information; (ii) knowledge on the structure, purposes, and functions of rabies surveillance; (iii) data collection, data management, and reporting methods; and (iv) surveillance attributes. For the assessment of certain items of surveillance attributes, participants were asked to document their answers on 10-point Likert scales, ranging from “not at all” to “great,” from “very unlikely” to “very likely,” from “very easy” to “very difficult,” or from “strongly disagree” to “strongly agree.” The use of the 10-point Likert scale aimed to obtain the extent to which health care and animal health staff had favorable or unfavorable attitudes toward rabies surveillance.

#### Simplicity

This consisted of three items. Participants provided their assessment on 10-point scales on the overall simplicity of rabies surveillance, standardized case definition of human rabies, and ease of completing standard report forms.

#### Flexibility

This consisted of two questions asking about the extent to which the case notification was integrated with other systems and the ease of the transition from using the paper-based to electronic case notification systems.

#### Acceptability

This consisted of four items and one indicator. Participants indicated their degree of agreement with the following items: rabies surveillance at both human and animal health facilities, the requirement of specimen collection, the reporting methods, and the willingness of staff in preparation and reporting data on rabies. Research staff also collected information on the percentage of specimens collected from human rabies cases over time.

#### Data Quality

Before the interview ended, all interview answer sheets had been checked for any missing information to ensure the validity of the data. In our surveillance review, this part had two 10-point Likert questions asking about the frequency of data sharing between the health and veterinary health sectors and the frequency of user-owned data checking for timeliness and completeness.

#### Timeliness

This was assessed using two indicators: (1) the number of late reports of PEP vaccination in 2017 collected during site visits at PCDCs or district health centers in the selected areas and (2) the percentage of human rabies cases that were entered by a hospital's staff into the national electronic case notification system, commencing 2016, within 2 days after being diagnosed with rabies through comparing data entry dates and dates of diagnosis on the case notification reports available over 2016–2018.

#### Usefulness

This was assessed using two indicators: (1) the percentage of participants reporting the use of surveillance data for rabies control and prevention measures and (2) the percentage of participants reporting the use of surveillance data for making a decision on prevention control policies or programs.

#### Stability

This part had two indicators: (1) the percentage of participants experiencing computer outages in the past month before being interviewed and (2) the percentage of continuous implementation of rabies surveillance in case of a budget cut in the future.

Human rabies cases have typical symptoms after bites, scratches, and open wounds exposed to the saliva of a rabid animal (rabid dogs in most cases in Vietnam) and are virtually always fatal 1–2 weeks after the onset of the clinical symptoms. The disease characteristics are usually typical and make the disease rarely misdiagnosed. Thus, an assessment of the positive predictive value attribute could not be essential. In Vietnam, all human rabies cases need to be reported to the preventive medicine system, so we did not assess the representativeness of the rabies surveillance system.

### Data Analysis

We created a timeline and structure summarizing the major guideline of rabies surveillance over time and the rabies case notification and routine PEP programmatic reporting systems. We calculated the incidence of human rabies per 10 million population for the period 2009–2018 and analyzed demographic, epidemiological, and clinical characteristics of human rabies cases whose notification forms were available for the analysis (2012–2018).

To analyze participants' responses in the 10-point Likert scale format, we removed neutral responses (score 5) to better distinguish between participants with favorable (score 6–10) and unfavorable attitudes (score 0–4) toward rabies surveillance. As such, denominators varied across the attributes evaluated in this report. We used the chi-square test or Fisher's exact-test to compare the proportion of an attitude/indicator in the animal health sector with the proportion of that attitude/indicator in the human health sector. Data were analyzed at PIHCMC using Stata 14 (StataCorp, TX).

## Results

### Epidemiology of Human Rabies in Southern Vietnam

**Figure 1:**
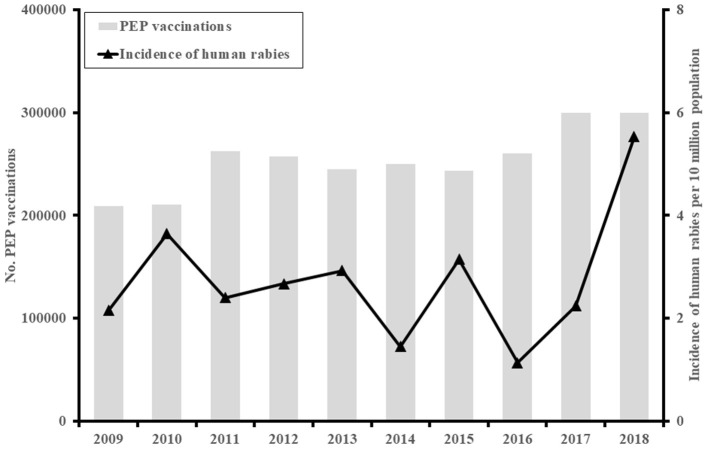
The incidence of human rabies cases (*N* = 94) and post-exposure prophylaxis (PEP) vaccinations in Southern Vietnam, 2009–2018.

In total, 94 cases of human rabies were reported from 17 of 20 provinces in the south between 2009 and 2018, with an average of nine cases recorded annually (2.7 cases per 10 million population). The highest number of cases was seen in 2018 (20 cases, 5.5 cases per 10 million population) (Figure 1).

Of the 94 human rabies cases, characteristics were available for 67 (71%) (Table 1). The majority (48, 72%) of cases were male and 20 years old or older (55, 82%). Almost all cases reported dog bites with wounds, mostly on their hands or arms (24, 36%) or lower limbs (14, 21%). Sixty-two cases (93%) had exposure category class III, which was single or multiple transdermal bites or scratches (6), but none received PEP. Three quarters (51, 76%) were not aware of the danger of rabies. About one in eight cases (15%) sought traditional medicine. Of 67 cases, specimens were collected at hospitals from 20 patients (30%) after the onset of clinical symptoms; 13/20 (65%) specimens were tested positive for rabies, and none of these patients received PEP after dog bites or scratches.

**Table 1:** Characteristics of 67 human rabies cases, 2012–2018.

Characteristics^*^	**Number of patients**	**Percent (%)**
**Age**		
0–9	6	9
10–19	6	9
20–29	10	15
30–39	10	19
40–49	13	18
50 and above	12	30
**Gender**		
Male	48	72
Female	19	28
**Animal vector**		
Dog	63	94
Cat	2	3
Others	2	3
**Exposure type**		
Bite	62	93
Others	5	7
**Exposure category**		
II	2	3
III	62	93
Unknown	3	4
**Wound position**		
Head/face	7	10
Arm/hand	24	36
Lower limb	14	21
Trunk	1	2
Others	21	31
**PEP immunization**		
No	67	100
Yes	–	–
**Seek traditional medicine**		
No	57	85
Yes	10	15
**Awareness of rabies**		
No	51	76
Yes	16	24
**Rabies testing (*****N*** **=** **20)^†^**		
Negative	7	35
Positive	13	65

* *Notifications of 32 cases of human rabies during 2009–2011 were unavailable for this review*.

† *Specimens were collected since 2014 (20/46). PEP, post-exposure prophylaxis*.

### Rabies Surveillance and Its Operations

**Figure 2:**
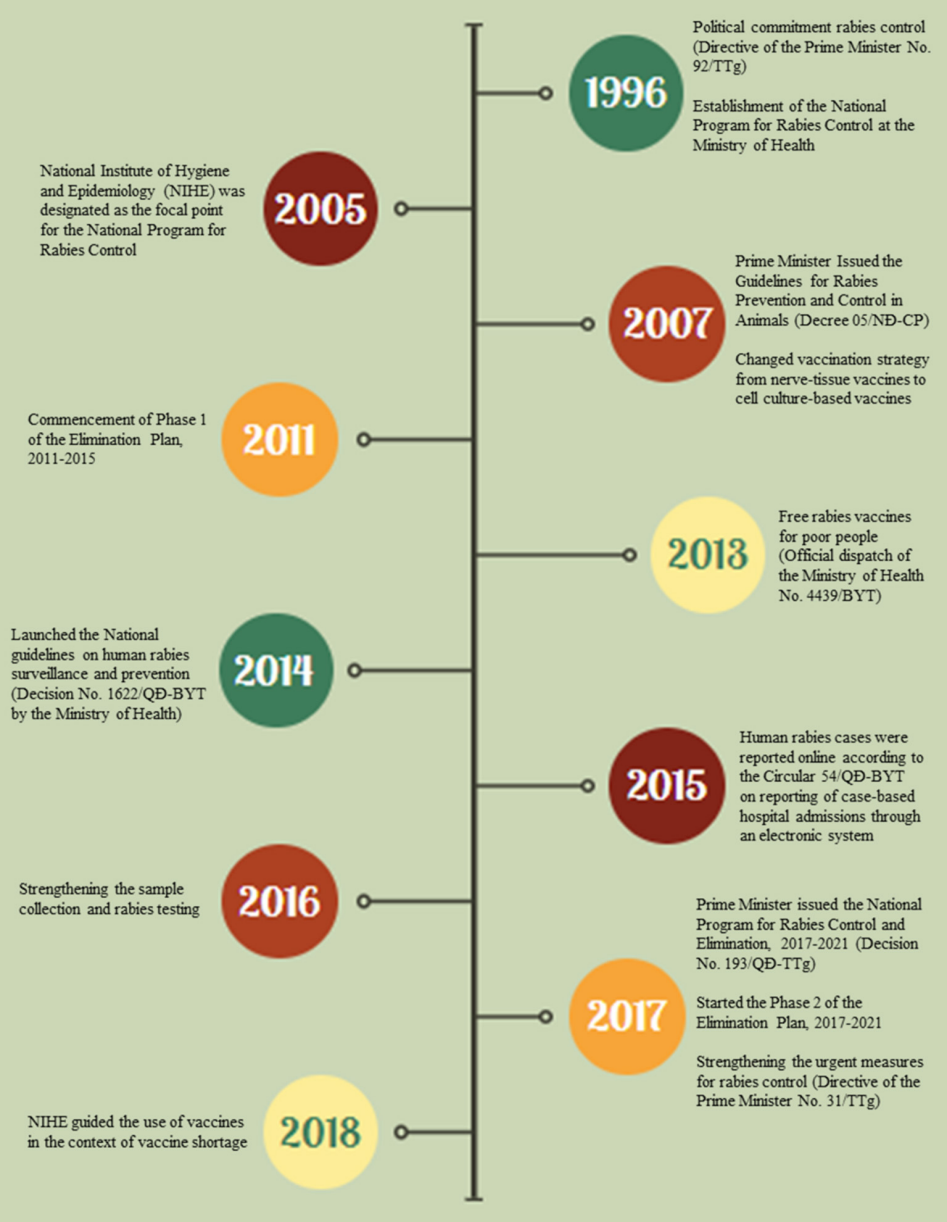
Timeline of key rabies surveillance and control policies in Vietnam.

Guidelines for rabies surveillance have evolved in Vietnam over the past two decades (Figure 2). Four methods have been used in Vietnam to monitor the epidemiology of human and canine rabies. These methods were outlined in internal technical materials prepared by the national program for rabies control before 2014 and the national guideline on human rabies surveillance and prevention by the Ministry of Health thereafter (Decision No. 1622/QÐ-BYT) (7). Reporting of these data occurred passively and included the population size of domestic dogs and cats, vaccination coverage among domestic animals and animal-bite victims, clinical notifications, and laboratory-confirmed cases of human and animal rabies.

#### Population Size of Domestic Animals

There have been no national standard methods for estimating the population size of domestic dogs and cats in Vietnam. In 2007, Vietnam issued its first national guideline for rabies prevention and control in animals (Decree 05/NÐ-CP by the Prime Minister), which stipulated that owners must register their dogs with the local government at the commune where they reside. However, dog registries for owners are still very rare in most areas of Vietnam. Only two of our five study provinces had estimates for the population size of domestic animals: there were ~107,000 dogs in Dong Nai and more than 220,000 dogs in HCMC, indicating dog ownership rates of 97 and 26 dogs per 1,000 persons, respectively. Of note, these estimates used the number of dogs receiving vaccination, which was manually reported by district animal stations or computerized and shared directly to stakeholders. Thus, this is certainly an underestimated number.

#### Vaccination Coverage

Vaccinating dogs for rabies is one of the prioritized activities for animal health sectors included in two medium-term elimination plans for the periods 2011–2015 and 2017–2021 and the first national strategy issued in 2017. Each province annually receives an allotment of rabies vaccines needed to vaccinate at least 90% of dogs and cats. The actual rabies vaccination coverage in domestic animals remains unknown and varied substantially across settings, as found in the study districts [mean coverage 55.6% (25,546/46,317), range: 8.5% (1,000/11,749)−99.3% (9,553/9,620)].

Used as the key measure for reducing the risk of developing rabies following a probable rabies exposure, rabies PEP services for exposed persons are available across the country, and the use of PEP was guided in detail and was consistent across technical materials and guideline over the past two decades. Data on PEP vaccination, however, have been systematically collected in Vietnam since 2014 as per Decision No. 1622/QÐ-BYT by the Ministry of Health (7). Immunization clinics at district levels or above are asked to report these data monthly (Supplementary Figure 1A). In Southern Vietnam, there were ~300,000 administrations of PEP to human victims of animal bites in 2018, a nearly 50% increase compared to that in 2010 (Figure 1).

#### Clinical Notifications

There are no formal definitions of clinical rabies for dogs or cats in Vietnam. Owners are requested to report any dogs or cats with suspected rabies to the veterinary officer at the commune where they live. These dogs or cats are quarantined for 14 days, and if they continue to show signs of rabies, their heads are collected for rabies testing.

Unlike canine rabies, furious and paralytic (or dumb) forms of rabies in humans were clearly defined in technical materials or the surveillance guideline. In this surveillance guidance, a person is diagnosed as a clinical human rabies case if he or she manifests acute encephalitis with agitated behaviors and fear of water (hydrophobia), of wind (aerophobia), and of exposure to light (photophobia) (the furious form) or develops gradual paralysis after the bite or scratch of a dog or cat (the paralytic form); the patient usually progresses to coma and finally death after 7–10 days. For more than 40 years, cases of human rabies have been a legislatively notifiable infectious disease, and health care facilities are legally required to report any cases diagnosed to the appropriate officer of the preventive medicine system in the same area where the case was detected with 48 h. Initially, manual reports of cases in human were conducted and then replaced by electronic ones since 2015 (Supplementary Figure 1B).

#### Laboratory-Confirmed Cases

Laboratory confirmation of rabies requires detection of the virus from heads of dogs or cats and cerebrospinal fluid, saliva (oral fluid), or biopsy skins collected from clinically suspected rabies patients. Despite having detailed laboratory guidance shared widely since 2010, few specimens were collected and submitted to regional institutes for rabies testing. The National Institute of Hygiene and Epidemiology enhanced rabies surveillance by requesting a collection of specimens and laboratory investigation from all reported rabies cases in humans (Figure 2). Sequences of rabies isolates from human cases have been limitedly available in Vietnam (8).

### Evaluation of Surveillance Attributes and Usefulness

#### Sample Characteristics

From June to November 2018, we interviewed a total of 92 human health staff and 53 animal health staff who played roles in the administration of rabies vaccines for dogs or cats, PEP for exposed persons, and detection, notification, and response to canine or human rabies cases. They came from 11 provincial-level and 19 district-level facilities.

Their characteristics are summarized in Supplementary Table 1. Over a half of the participants (84/144, 58%) were male, and their median age was 38 years (range: 25–58 years). Three quarters (108/145, 74%) had completed a university degree, and 59% (82/140) had 10 or more years of work experience. Only 2% (3/145) of participants had received a rabies vaccination. One third (54/145, 37%) had ever attended a training on rabies. About 44% (63/145) of participants had moderate to high knowledge of the rabies surveillance guideline, including the objectives, domains, case definitions, specimen requirement, and reporting methods of the surveillance [percentage range: 61% (88/145)−97% (141/145)]. Apart from the interviews, we also reviewed data from 192 monthly reports of PEP vaccination and 53 reports of rabies vaccines for dogs and cats.

#### Attributes of Rabies Surveillance

Table 2 presents the results of the assessment of attributes of rabies surveillance in five provinces in Southern Vietnam.

**Table 2:** Attributes of rabies surveillance.

**Attribute**	Participants' favorable attitudes^*^/surveillance indicator	***N***	**Percent (%)**
Simplicity	Rabies surveillance was simple	87	61^†^
	Case definitions of human rabies were standardized^‡^	73	89
	Monthly PEP reporting forms of rabies surveillance are easy to complete^‡^	53	24
Flexibility	Able to integrate rabies case notifications into the general reporting system for notifiable infectious diseases	89	84
	Able to make transition from using the paper-based to electronic case notification systems for rabies^‡^	55	42
Acceptability	Deployment of rabies surveillance is acceptable at human health and animal health sectors	115	84
	Reporting methods: monthly for PEP vaccination and casual reports for cases of human rabies	112	90
	Voluntary involvement in reporting rabies data	114	87
	Agreed with the requirement of specimen collection of human rabies cases^‡^	68	87
	Percentage of specimens collected from human rabies cases^‡^	67	30
Data quality	6-month data sharing between human health and veterinary health	98	83
	Checking for timeliness and completeness of data	92	83
Timeliness	Percentage of human rabies cases reported within 48 h after diagnosis^‡^	29	7
	Percentage of on-time reports of animal-bite victims receiving PEP^‡^	192	58
Usefulness	Percentage of participants who witnessed the use of surveillance data for control and prevention activities	144	39^†^
	Percentage of participants who witnessed the use of surveillance data for policy advocacy or programming	145	21^†^
Stability	Percentage of experience of system outrage or damage in the past month^‡^	86	22
	Percentage of continuous implementation of rabies surveillance in case of budget cuts^‡^	144	75

*N indicates the number of participants or forms; PEP, post-exposure prophylaxis*.

* *Participants' attitude was distinguished as favorable (score 6–10) and unfavorable (score 0–4) toward rabies surveillance*.

† *Differences in attitudes between human health and animal health were statistically significant*.

‡ *Data were obtained only from health care workers*.

##### Simplicity

Sixty-one percent of participants indicated that overall rabies surveillance was simple. Significantly more participants from animal health sectors than from human health sectors indicated that, overall, rabies surveillance was simple [79% (27/34) vs. 49% (26/53), *p* = 0.005]. Only 77% (41/53) of health workers agreed that the monthly PEP routine reporting forms were easy to complete. Almost all interviewed (65/73, 89%) favored the standardized case definitions of human rabies.

##### Flexibility

Possible integration of rabies case notifications into the general reporting system for notifiable infectious diseases was stated by 84% (75/89) of participants. Forty-two percent (23/55) of data staff and managers surveyed showed a favorable attitude toward the change from the paper-based to electronic case notification systems for rabies.

##### Acceptability

The majority of participants agreed that rabies surveillance should be done at both human health and animal health sectors (97/115, 84%) and that reporting methods should include the routine vaccination report and casual case notification (101/112, 90%). Most participants (99/114, 87%) considered surveillance activities as part of the responsibility of their organizations. Almost all (59/68, 87%) agreed on the collection of specimens from human rabies cases, but this was not commonly performed: 20 out of 67 cases (30%) identified during 2012–2018 were collected specimens for rabies testing.

##### Data Quality and Timeliness

The majority of participants expressed a favorable attitude toward the frequent 6-month data sharing between human health and animal health sectors (81/98, 83%) and the frequent user-owned checking forms for timeliness and completeness of data (76/92, 83%). Of note, there was a difference in the reported number of human rabies cases and the number of patients with A82.9 ICD10 extracted from the electronic hospital management system during field visits in the five provinces in 2017 (12 vs. 13 cases). This yielded a 92% completeness of case notifications. Timely reports were rare: only 7% (2/29) of human rabies cases identified in 2016–2018 and 58% (111/192) of monthly data on PEP vaccinations were reported timely, defined as a notification within 2 days of a clinical or laboratory-confirmed case of human rabies. Hard copies of 53 reports on rabies vaccination in animals from animal health facilities were mostly unavailable for assessing the completeness and timeliness of reporting data.

##### Usefulness

Thirty-nine percent (56/144) and 21% (31/145) of participants reported observing the use of surveillance data for control and prevention activities and policy and advocacy activities, respectively. A significantly higher proportion of participants from animal health sectors [57% (30/53) and 40% (21/53)] than from health sectors [29% (26/91) and 11% (82/92)] reported these experiences (*p* = 0.001 and *p* < 0.001, respectively). Measures ranged from outbreak monitoring to data reporting, development of risk communications, and free rabies vaccination programs for animals and exposed persons and a call for the involvement of the local government authority and its departments and the community in rabies control and prevention.

##### Stability

Over one in five participants (22%, 31/144) reported an experience of computer outages in the previous month before being interviewed. Three quarters (108/144) indicated that rabies surveillance will continue to be implemented even when the budget is cut.

## Discussion

The low annual incidence of human rabies reported in our surveillance review may indicate the low-level transmission of rabies from animals to humans in Southern Vietnam. This is as low as in Thailand and much lower than those seen in other Asian countries (9) and lower than the incidence found in the Northern Vietnam (10). Large-scale implementation of rabies vaccination programs for domestic dogs over an extended period of time and standard, simplified, and widely accessible PEP regimens (11), in conjunction with greater public awareness about rabies associated with health promotion programs and economic growth, may have contributed to this low incidence of human rabies. However, we found frequent transmission of rabies from domestic dogs to humans in Southern Vietnam in recent years. Improvements in the implementation of rabies surveillance over the evaluation period may explain the increase in notifications. Despite this, our finding, together with the low completion rates of PEP among animal-bite individuals and patients found in a previous study (10), indicates a significant challenge for this region to control canine rabies by 2021 and keep track of the progress toward elimination of dog-transmitted human rabies. Addressing this issue will require greater political will and involvement of different levels of the government and public sectors, larger resources, better strategic planning, and continued surveillance, response, and prevention programs of both human health and animal health sectors. The target population is adults due to their predominance among rabies patients and animal-bite victims as had been seen in other Asian countries (12, 13).

We found evidence that rabies surveillance was simple, flexible, widely acceptable, and stable at different levels of human health and animal health systems. Possible reasons for these strengths were the presence of the national rabies strategy and technical guidelines, the availability of concurrent structural systems of human health and animal health, the public health significance of canine rabies and human rabies, and the prolonged public health interventions to control rabies and eliminate rabies in animals and humans.

Apart from the aforementioned strengths, we found several potential target areas for improvement. In strategic planning and programming, although continuing the strategies for vaccinating domestic dogs and cats against rabies is highly important for control and prevention of the spread of rabies to humans, there was a lack of methods and reliable estimates for the population size of domestic dogs and cats in the community. Program managers are thus unable to design a proper management system for domestic dogs and cats, identify risk areas, develop strategic immunization plans, understand where new cases might be occurring, and stop transmission of the disease. The capture–recapture and sight–resight methods that have been used in many countries, like Thailand, India, and Haiti, for estimating the population size of wild animals may be adapted to address this issue (14–16). Another challenge is data usage for short-term prevention activities and long-term strategic program decision making and policy advocacy, particularly among staff from health sectors, as a low percentage of them were aware of turning obtained surveillance data into public health actions. Addressing this issue is difficult but important for advocating, planning, and implementing control and prevention programs that work.

In operation, we frequently observed unfavorable attitudes toward the reporting forms of rabies surveillance. Observations that several individual pieces of information are required and extracted manually from immunization logbooks at clinics for a routine paper-based report of PEP vaccination and collected from a case of human rabies support this attitude. The lack of appropriate training as reported among our participants is another possible reason for the unfavorable attitude. This may also affect the low percentage of specimen collection from human rabies cases due to the poor knowledge about the possible types and sufficient volume of specimens for testing, procedures for collection, storage and transportation, and laboratory locations. Hands-on training courses and regular site visits with on-the-job training are needed to enhance the completion of data collection and management.

In addition to the unsatisfactory completion rates, the timelines of monthly PEP reporting and the 48-h notification of a case of human rabies by health authorities were disappointing, especially the case notification in contrast to the 92% completeness rate of case notification of this surveillance. Delayed reporting may be caused by the use of a paper-based reporting system and the thought that a prompt investigation and reporting of this almost fatal infectious Class B disease may not be necessary. Vietnam has enforced its case notification and immunization systems by requesting real-time reporting of certain notifiable infectious diseases using the electronic communicable disease system (eCDS) since 2015 and of immunization information using the national immunization information (NIIS) from 2019 (17). Encouraging and monitoring the use of eCDS for rabies case notifications at hospitals and expanding the NIIS for routine PEP at all health levels could improve the timeliness of reporting. Such improvement, especially case notification, is crucial for prompt case investigations to identify other probable rabies exposures referred for PEP and implementation of quarantine of potentially rabid animals and removal of rabid animals in the area where a human rabies case occurred.

Our evaluation has several limitations. First, a non-random convenience sampling method was used to enroll participants with variations in background characteristics, age, duration of work, and experience in 10 districts in five provinces. The degree to which these people were representative of the entire staff involved in rabies surveillance in Southern Vietnam is unknown. Second, as other studies that entirely depend on face-to-face interviews, there are opportunities for the occurrence of social desirability biases, resulting in misclassification of favorable and unfavorable attitudes toward surveillance attributes. Future assessments of surveillance attributes as per the U.S. CDC guideline with a larger, more representative sample size are required to limit the uncertainty and biases around the survey responses. Third, hard copies of laboratory results and reports from animal sectors are unavailable.

Our findings suggest a low transmission of rabies from animals to humans in Southern Vietnam. Despite the common views of the simplicity, flexibility, acceptability, and stability of rabies surveillance, simplifying the reporting forms, training the staff, and improving the timeliness of reporting and data usage are highly recommended. Our finding of a potential increased incidence of human rabies warrants further inspections.

## Data Availability Statement

The raw data supporting the conclusions of this article will be made available by the authors, without undue reservation.

## Ethics Statement

The studies involving human participants were reviewed and approved by Institutional Review Board at the Pasteur Institute in Ho Chi Minh City. Written informed consent for participation was not required for this study in accordance with the national legislation and the institutional requirements.

## Author Contributions

QP led the design of the study, collected the data, performed all statistical analyses, interpreted the data, and wrote the first draft of the manuscript. TPTN, QD, and HN collected and managed the data. LP, QL, and TVN assisted in the interpretation of findings, reviewed the manuscript, and contributed to the critical revision of the manuscript. LP and TVN sought for research grants and overviewed the study. All authors have approved the final version.

## Conflict of Interest

The authors declare that the research was conducted in the absence of any commercial or financial relationships that could be construed as a potential conflict of interest.
