# Supplementary material for: An Evaluation of the Rabies Surveillance in Southern Vietnam
Source: Front Public Health. 2021 Apr 29;9:610905. doi: 10.3389/fpubh.2021.610905 (PMC8116623; doi:10.3389/fpubh.2021.610905)

Supplementary Material

**Supplementary Table 1. Characteristics of participants.**

| **Characteristics** | **Human Health sector (N = 92)** | | **Animal health sector (N = 53)** | | **Overall**  **(N = 145)** | |
| --- | --- | --- | --- | --- | --- | --- |
|  | n | Percent | n | Percent | n | Percent |
| Age (median years) | 91 | 37 | 53 | 41 | 144 | 38 |
| Gender |  |  |  |  |  |  |
| Male | 42 | 46% | 42 | 79% | 84 | 58% |
| Female | 49 | 54% | 11 | 21% | 60 | 42% |
| Level |  |  |  |  |  |  |
| Provincial | 26 | 28% | 23 | 43% | 49 | 34% |
| District | 66 | 72% | 30 | 57% | 96 | 66% |
| Highest education level |  |  |  |  |  |  |
| High school/vocational school | 32 | 35% | 5 | 9% | 37 | 26% |
| Undergraduate | 39 | 42% | 40 | 75% | 79 | 54% |
| Postgraduate | 21 | 23% | 8 | 15% | 29 | 20% |
| Years of work experience |  |  |  |  |  |  |
| <5 | 20 | 23% | 1 | 2% | 21 | 15% |
| 5-9 | 19 | 22% | 18 | 34% | 37 | 26% |
| 10-15 | 16 | 18% | 11 | 21% | 27 | 19% |
| 16-19 | 4 | 5% | 2 | 4% | 6 | 4% |
| 20 or above | 28 | 32% | 21 | 40% | 49 | 35% |
| Received rabies vaccine series | 1 | 1% | 2 | 4% | 3 | 2% |
| Received training on rabies | 32 | 35% | 22 | 41% | 54 | 37% |
| Knew the rabies surveillance guideline | 37 | 40% | 26 | 49% | 63 | 44% |
| Knew the objectives of surveillance | 61 | 66% | 43 | 81% | 104 | 72% |
| Knew domains of rabies surveillance^*^ | 88 | 96% | 53 | 100% | 141 | 97% |
| Knew surveillance case definitions | 66 | 72% | 38 | 72% | 104 | 72% |
| Knew specimens for rabies diagnosis | 78 | 85% | 38 | 72% | 116 | 80% |
| Knew the rabies reporting system |  |  |  |  |  |  |
| Routine report | 22 | 24% | 35 | 66% | 57 | 39% |
| Urgent report | 70 | 76% | 18 | 34% | 88 | 61% |

*******In the present study, respondents were categorized as having the knowledge about major domains of the rabies surveillance system if he/she correctly answered one of the four following subjects of the surveillance, including *i*) human rabies cases; *ii*) exposed people; *iii*) suspected canine animals; and *iv*) an outbreak.

**Supplementary Figure 1. Notification of rabies cases and routine reports of post-exposure prophylaxis vaccination in Vietnam**


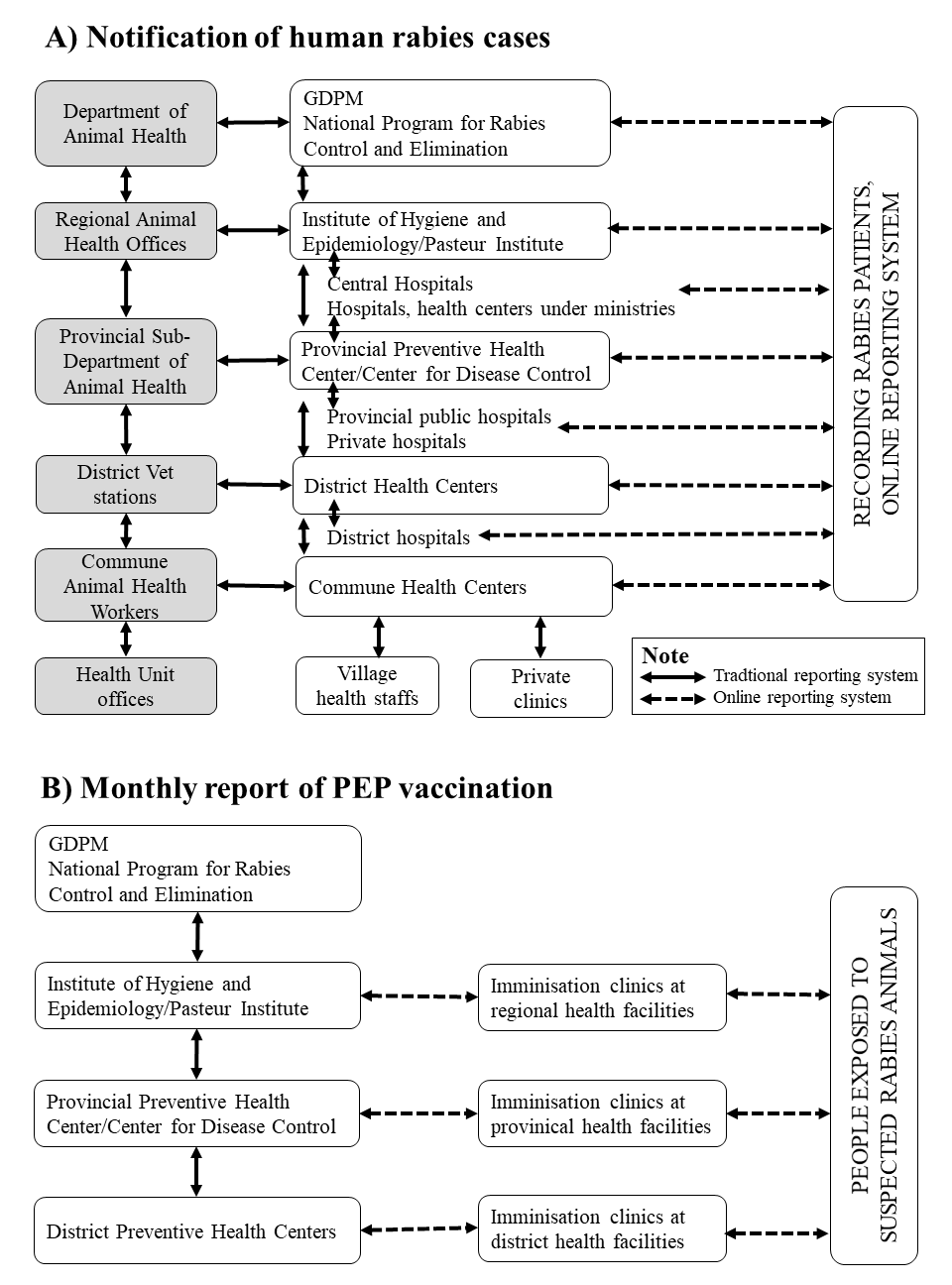

Supplement: Supplementary file 1 [file Data_Sheet_1.docx]
